# Asiatic Acid Attenuates *Salmonella typhimurium*-Induced Neuroinflammation and Neuronal Damage by Inhibiting the TLR2/Notch and NF-κB Pathway in Microglia

**DOI:** 10.3390/ijms27020602

**Published:** 2026-01-07

**Authors:** Wenshu Zou, Jianxi Li

**Affiliations:** 1 Jiangxi Province Key Laboratory of Traditional Chinese Medicine Pharmacology, Institute of Traditional Chinese Medicine Health Industry, China Academy of Chinese Medical Sciences, Nanchang 330115, China; zouwenshu080603@163.com; 2 Postdoctoral Fluxion Station, China Academy of Chinese Medical Sciences, Beijing 100700, China; 3 Key Laboratory of Veterinary Pharmaceutical Development of Ministry of Agriculture and Rural Affairs of China, Engineering and Technology Research Center of Traditional Chinese Veterinary Medicine of Gansu Province, Lanzhou Institute of Husbandry and Pharmaceutical Sciences of Chinese Academy of Agricultural Sciences, Lanzhou 730050, China

**Keywords:** iNOS, *Salmonella* infection, asiatic acid, microglia, TLR2/Notch

## Abstract

*Salmonella typhimurium* (*S.T*) infection of the central nervous system (CNS) induces severe inflammation, leading to elevated expression of inducible nitric oxide synthase (iNOS) in microglia. This process catalyzes excessive production of nitric oxide (NO), resulting in irreversible damage to neuronal mitochondria. Asiatic acid (AA) is a small molecule with neuroprotective potential; however, its ability to counteract nerve injury induced by *S.T* and the underlying mechanisms remain unclear. In this study, we established an *S.T*-infected mouse model (in vivo) and an *S.T*-stimulated microglial model using BV-2 cells (in vitro) and employed techniques including immunofluorescence (IF), Western blot, co-immunoprecipitation (Co-IP), and RNA extraction and quantitative reverse transcription PCR (RT-qPCR) to systematically evaluate the protective effects and mechanisms of AA. The results showed that pre-treatment with AA significantly reduced the expression of iNOS and the production of NO caused by *S.T* infection in mouse hippocampal tissue and BV-2 cells. Mechanistically, AA exerts its effects by inhibiting the upstream Toll-like receptor 2 (TLR2)/Notch and nuclear factor-κB (NF-κB) signaling axis. It interferes with the nuclear translocation of Notch and p65 proteins and their complex formation under *S.T* stimulation, thereby blocking downstream expression of iNOS and production of NO. This study reveals a novel mechanism by which AA alleviates infection-related neuroinflammation through targeting Notch-p65 interactions, providing a new theoretical basis for its clinical application.

## 1. Introduction

A total of 151 genetically related cases suspected to be linked to consumption of the implicated chocolate products have been reported across 11 countries [1]. Although *Salmonella* infections are typically mild and do not require treatment, children and elderly individuals are at greater risk for severe complications related to associated dehydration; meanwhile, Salmonella infection of the central nervous system (CNS) is possibly fatal to both humans and animals [2,3,4]. *Salmonella* infections are often associated with high mortality and complications, including serious neurological sequelae and high relapse rates [5]. Salmonella-infected mice develop characteristic neurological symptoms, such as head tilt and rotational behavior, accompanied by systemic inflammation [6]. *Salmonella* invades macrophages and subsequently disseminates throughout the body, including the liver, spleen, kidneys, and brain. Infected macrophages produce microbicidal compounds, such as reactive oxygen species (ROS) [6] and nitric oxide (NO) [7]. Multiple studies have demonstrated a strong association between NO and *Salmonella* infection. Restriction of inducible nitric oxide synthase (iNOS) production in macrophages by *Salmonella* pathogenicity island 2 (SPI2) promotes immune evasion and facilitates bacterial colonization [8]. However, inhibition of iNOS protects the brain from damage. Previous studies have shown that iNOS inhibition enhances the antioxidant capacity of brain parenchymal cells and reduces apoptosis-associated histological damage in the rat brain [9,10]. Additionally, suppression of iNOS expression reportedly promotes apoptosis in glioma stem cells, thereby achieving therapeutic antitumor effects [11,12]. Excessive NO production activates upstream Notch and NF-κB signaling pathways, which promote the release of additional inflammatory mediators and further exacerbate neuroinflammation [13]. Therefore, regulation of iNOS expression represents an important component in strategies to prevent *Salmonella* infection-associated neurological injury.

As an upstream regulator of iNOS, nuclear translocation of the Notch intracellular domain (NICD) promotes iNOS expression and plays an important role in the CNS [14,15]. After translocation into the nucleus, NICD binds to CSL (CBF-1, Suppressor of Hairless, Lag) transcription factors, activating downstream target genes such as *Hes1* and *Hes5* [16]. Several studies reported that iNOS enhances Notch signaling by inducing Hes1 expression. Multiple studies have demonstrated that regulation of the Notch pathway in CNS protection is closely associated with microglia [17]. Inhibition of Notch signaling and iNOS expression promotes microglial differentiation toward the anti-inflammatory M2 phenotype [18]. Neuronal injury in ischemic stroke is associated with increased apoptosis, proinflammatory leukocyte infiltration, and microglial, accompanied by extensive nuclear translocation of the NICD [19]. In contrast, M2-type microglia attenuate neuronal apoptosis through activation of Notch1 [20]. Collectively, activation of the Notch signaling pathway may exert either protective or deleterious effects on microglia and the nervous system, depending on the pathological context. Additionally, as a canonical inflammatory pathway, Notch signaling regulates downstream NF-κB–mediated cell death and inflammatory responses. Inhibition of γ-secretase has been shown to prevent increased NF-κB expression in microglia and to block NICD nuclear translocation in activated microglia [21]. Other studies have indicated that interactions between p65 and NICD are critical for NF-κB pathway activation in response to interleukin (IL)-1β and tumor necrosis factor (TNF)-α [22]. Furthermore, NF-κB–induced inflammatory mediators and iNOS interact to amplify inflammatory responses [23]. Toll-like receptor 2 (TLR2) is expressed on various cell types; it activates downstream Notch and NF-κB signaling pathways to facilitate responses to pathogenic microbial invasion. However, there remains uncertainty concerning whether *Salmonella* activates TLR2 and the downstream Notch and NF-κB pathways, as well as the mechanistic role of this activation in CNS regulation.

The therapeutic benefits of *Centella asiatica* in anxiety, cognitive impairment, and memory decline are largely attributed to its active compound, asiatic acid (AA) [24]. As a well-characterized neuroprotective pentacyclic triterpenoid, AA counteracts cerebral ischemia–induced mitochondrial damage and blood–brain barrier hyperpermeability [25]. Endogenous neurotoxin–related cognitive impairment and oxidative stress in the brain have also been alleviated by AA treatment [26]. AA protects neurons from inflammatory injury by inhibiting the production of ROS from mitochondria, and downregulating the expression of IL-6, IL-1β and TNF-α in microglia [27,28]. AA also alleviates diabetic retinopathy by inhibiting microglial activation [29]; however, its effects and underlying mechanisms in *Salmonella*-induced CNS injury remain unclear. In the present study, we found that AA reduces *S.T*–induced mitochondrial damage, microglial activation, and iNOS expression in the hippocampus, through a mechanism associated with modulation of the TLR2/Notch signaling pathway.

## 2. Result

### 2.1. AA Reduces iNOS Expression to Suppress S.T-Induced Overproduction of NO in the Hippocampus

**Figure 1:**
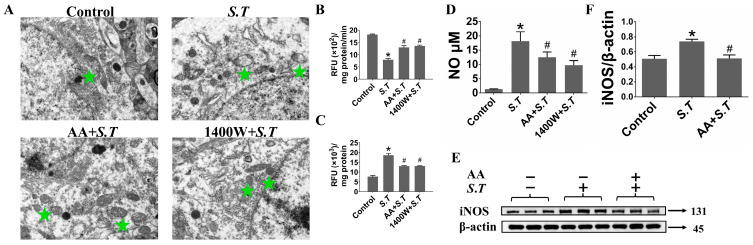
AA regulates *S.T*-induced NO secretion in hippocampus by inhibiting iNOS expression. (**A**) Representative TEM images showing the mitochondria in the hippocampus of mice. The green pentagram refers to the mitochondria (Micrograph at 7000× magnification). (**B**) The BboxiProbe^®^ R02 oxygen fluorescent probe was used to detect mitochondrial OCR in the hippocampus of mice. (**C**) Rhodamine 123 probe was used to detect mitochondrial membrane potential collapse in the hippocampus of mice. (**D**) The expression level of NO is in the hippocampus of mice was analyzed by Griess reaction (*n* = 3, * *p* < 0.05, compared with control; # *p* < 0.05, compared with *S.T*). (**E**) The protein level of iNOS in the hippocampus of mice was analyzed by Western blot. (**F**) Grayscale values of iNOS protein expression in the mouse hippocampus (*n* = 3, * *p* < 0.05, compared with control; # *p* < 0.05, compared with *S.T*).

Previous studies have shown that NO inhibits mitochondrial respiration and disrupts neuronal metabolism in the hippocampus [30,31]. To evaluate the impact of *S.T* on the hippocampus after blood–brain barrier penetration, we harvested hippocampal tissues and examined them via transmission electron microscopy (TEM). Extensive mitochondrial damage was observed in the hippocampi of *S.T*-infected mice (Figure 1A). To further assess mitochondrial function, we measured oxygen consumption rate (OCR) and mitochondrial membrane potential in mouse brain tissue. Results indicate that mitochondrial OCR is significantly reduced in the hippocampi of mice stimulated with *S.T* (Figure 1B). Similarly, mitochondrial membrane potential collapse also occurred following *S.T* infection (Figure 1C). Subsequently, levels of NO and its inducible enzyme, iNOS, were assessed in the hippocampus; both levels were significantly increased after *S.T* infection (Figure 1D,E). Pretreatment with the iNOS inhibitor 1400W substantially reduced NO production and mitochondrial dysfunction in the hippocampus (Figure 1A–C), indicating that *S.T* infection increased NO production through iNOS upregulation and thus induced mitochondrial injury. AA pretreatment alleviated *S.T*-induced mitochondrial dysfunction (including mitochondrial damage, decreased mitochondrial OCR and mitochondrial membrane potential collapse), while simultaneously reducing NO production and inducible iNOS expression in the hippocampi of mice (Figure 1). Collectively, these findings indicate that AA suppresses overproduction of NO by reducing iNOS expression in the hippocampus, attenuating *S.T*-induced neural injury.

### 2.2. AA Inhibits S.T-Induced Activation of the NF-κB Pathway in the Hippocampus

**Figure 2:**
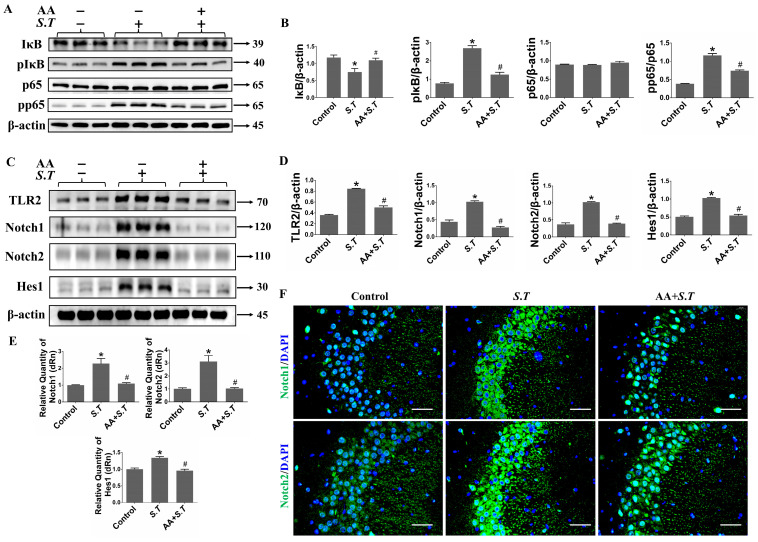
AA inhibits *S.T*-induced activation of the NF-κB and TLR2/Notch pathway in hippocampus. (**A**) Western blot to detect NF-κB protein levels in the hippocampus of mice with *S.T* stimulation and AA pretreatment. (**B**) Grayscale values of NF-κB pathway protein expression in mice hippocampi (*n* = 3, * *p* < 0.05, compared with control; # *p* < 0.05, compared with *S.T*). (**C**) Western blot to detect TLR2/Notch protein levels in the hippocampus of mice with *S.T* stimulation and AA pretreatment. (**D**) Grayscale values of TLR2/Notch pathway protein expression in mice hippocampi (*n* = 3, * *p* < 0.05, compared with control; # *p* < 0.05, compared with *S.T*). (**E**) qPCR detection of *Notch1*, *Notch2* and *Hes1* mRNA expression in *S.T*-challenged or AA pretreatment hippocampus (*n* = 3, * *p* < 0.05, compared with control; # *p* < 0.05, compared with *S.T*). (**F**) Representative immunohistochemistry (IHC) images showing the Notch1 and Notch2 protein levels in mice hippocampi (scale = 100 μM).

To evaluate the effects of excessive NO, we examined the NF-κB pathway, which is closely associated with NO signaling. Activation of the NF-κB pathway is primarily characterized by nuclear translocation of p65 to promote inflammatory gene transcription; phosphorylation of its inhibitor, IκB, leads to IκB degradation via E3 ubiquitin ligases [32]. Western blot analyses revealed increased IκB phosphorylation in the hippocampus of *S.T*-infected mice compared with controls, thereby facilitating p65 phosphorylation (Figure 2A,B). Consistent with these findings, phosphorylation and nuclear translocation of p65 were substantially increased in the hippocampus after *S.T* infection (Figure 2A,B). Pretreatment with AA significantly reduced *S.T*-induced phosphorylation of IκB and p65 and prevented IκB degradation (Figure 2A,B). In contrast, total p65 protein levels remained unchanged after *S.T* infection and AA pretreatment (Figure 2A,B). These observations are consistent with findings by Li et al., who demonstrated that total p65 levels are conserved during NF-κB activation; a fraction of p65 transiently translocases to the nucleus for phosphorylation before returning to the cytoplasm [33]. Collectively, the results indicate that AA suppresses NF-κB pathway activation induced by *S.T* infection.

### 2.3. AA Inhibits S.T-Induced Activation of the TLR2/Notch Pathway in the Hippocampus

As a cell surface receptor, TLR activates the downstream NF-κB pathway. Blocking TLR specifically inhibits NF-κB dependent high expression of iNOS [34]. To further elucidate the mechanisms by which *S.T* regulates iNOS expression, we assessed TLR2 expression on the cell surface. As expected, TLR2 expression was significantly increased after *S.T* infection compared with controls (Figure 2C,D). Pretreatment with AA greatly decreased *S.T*-induced TLR2 upregulation (Figure 2C,D), indicating that TLR2 is a key target of AA in protection against *S.T* infection.

The Notch signaling pathway is speculated to play an important role in the initiation of inflammatory responses and to function as a downstream signaling mediator of TLR2 in transmitting signals to the NF-κB pathway [16,35]. To clarify the involvement of the Notch pathway in *S.T* infection, Western blot, immunohistochemistry, and qPCR were performed to assess protein and mRNA expression patterns of Notch pathway components. The Notch intracellular domains (NICDs) of Notch1 and Notch2 were substantially increased in the hippocampi of *S.T*-infected mice compared with controls (Figure 2C,D,F). Consistent with these findings, qPCR analysis demonstrated elevated Notch1 and Notch2 mRNA expression levels in *S.T*-stimulated hippocampi (Figure 2E), indicating activation of the Notch pathway after *S.T* infection. Upon activation, Notch receptors are cleaved by γ-secretase, releasing the NICD, which translocases to the nucleus to activate target genes (e.g., *Hes1*) through interactions with CSL transcription factors [36]. Compared with findings in control samples, *Hes1* mRNA and protein levels were significantly increased in the hippocampus after *S.T* infection (Figure 2C–E). Both Western blot and immunohistochemistry analyses demonstrated that AA pretreatment reduced protein expression levels of Notch1, Notch2, and Hes1 in the hippocampi of *S.T*-infected mice (Figure 2C,D,F). Consistent with these results, qPCR analysis showed that AA pretreatment decreased Notch1, Notch2, and Hes1 mRNA expression levels in the hippocampus (Figure 2E). Collectively, our findings indicate that AA pretreatment suppresses *S.T*-induced activation of the TLR2/Notch signaling pathway in the hippocampus.

### 2.4. S.T Infection Increase NO via iNOS in Microglia

**Figure 3:**
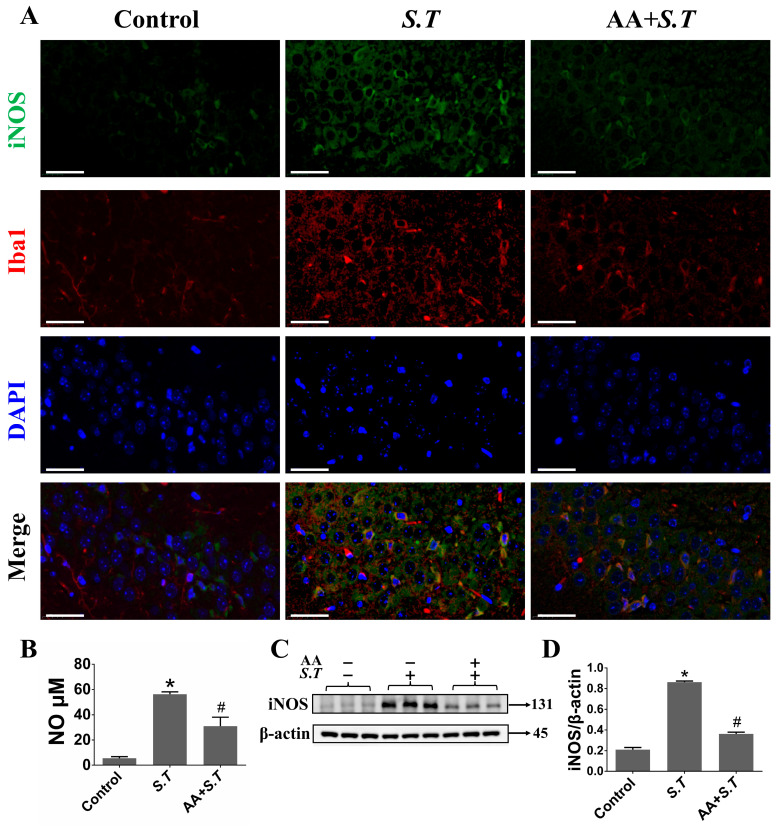
AA inhibits excessive NO in microglia caused by *S.T*. (**A**) Representative IHC images showing the iNOS and Iba1 protein levels in mice hippocampi (scale = 40 μM). (**B**) NO content in the microglia after *S.T* infection and AA pretreatment (*n* = 3, * *p* < 0.05, compared with control; # *p* < 0.05, compared with *S.T*). (**C**) The protein level of iNOS in the microglia after *S.T* infection and AA pretreatment was analyzed by Western blot. (**D**) Grayscale values of iNOS protein expression in the microglia (*n* = 3, * *p* < 0.05, compared with control; # *p* < 0.05, compared with *S.T*).

In the brain, neurons could be damaged by damaged by NO released from the activated microglial [37]. To demonstrate whether *S.T*-induced iNOS is associated with microglia, we localized iNOS^+^ cells and microglia in mouse brain sections by IHC. The results demonstrated that microglia are activated and a large number of iNOS^+^ cells accumulated near microglia after *S.T* infection compared with control (Figure 3A). The number of iNOS^+^ cells challenged by *S.T* decreased in the vicinity of microglia after pretreatment with AA (Figure 3A). Then, we measured NO levels in the supernatants of microglia cultures. The results showed that NO was elevated after *S.T* infection compared with control, and decreased after AA pretreatment compared with *S.T* (Figure 3B). Meanwhile, we examined the iNOS expression in microglia. As we suspected, AA reduced the *S.T*-induced elevated iNOS expression (Figure 3C,D). These results demonstrated that AA alleviated *S.T*-induced hippocampal damage by decreasing NO expression in microglia.

### 2.5. AA Inhibits S.T-Induced Activation of NF-κB Pathway in BV-2 Cells

**Figure 4:**
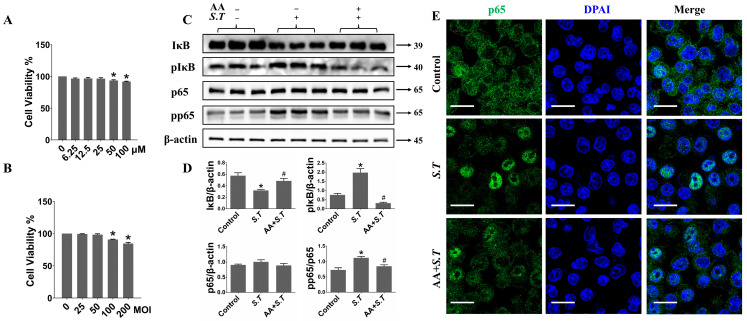
AA inhibits the activation of NF-κB pathway in BV-2 cells caused by *S.T*. (**A**,**B**) The effect of AA and *S.T* on the viability of BV-2 cells as measured using a CCK-8 assay. (**C**) Western blot to detect NF-κB pathway protein changes in *S.T* infection and AA pretreatment BV-2 cells. (**D**) BV-2 cells NF-κB pathway protein grayscale images (*n* = 3, * *p* < 0.05, compared with control; # *p* < 0.05, compared with *S.T*). (**E**) Confocal images demonstrate p65 nuclear translocation in microglia after *S.T* infection and AA pretreatment (scale = 20 μM).

Activation of the NF-κB pathway promotes inflammatory mediator release and represents a key indicator of macrophage activation. Previous results demonstrated that AA suppresses *S.T*-induced NF-κB pathway activation in vivo; however, its effects in vitro remained unclear. To determine appropriate concentrations of AA and *S.T* for experiments in microglia, we assessed cell viability across varying concentrations and exposure durations using the cell counting kit (CCK)-8 assay. Treatment with AA at concentrations ranging from 0 to 25 μM for 48 h did not significantly affect cell viability (Figure 4A). Next, we found that infection with *S.T* at MOI 100 for 24 h resulted in a significant reduction in cell viability (Figure 4B). Based on these findings, *S.T* infection at MOI 100 for 24 h was used to establish the BV-2 cells infection model. Western blot analyses revealed a marked decrease in IκB expression and concomitant increases in phosphorylated IκB (p-IκB) and phosphorylated p65 (p-p65) levels in *S.T*-infected BV-2 cells compared with controls (Figure 4C,D). After NF-κB activation, nuclear translocation of p65 enables target DNA sequence binding to exert downstream effects [38]. Confocal microscopy demonstrated pronounced nuclear translocation of p65 after *S.T* infection (Figure 4E). Pretreatment with AA inhibited p65 nuclear translocation and reduced IκB phosphorylation in *S.T*-stimulated BV-2 cells (Figure 4). As a canonical inflammatory signaling pathway, NF-κB plays a central role in BV-2 cell activation. The observed inhibition of NF-κB pathway activation by AA in microglia underscores its potential role in preventing *S.T*-induced central nervous system injury.

### 2.6. AA Regulates TLR2/Notch Expression in S.T Stimulated BV-2 Cells

**Figure 5:**
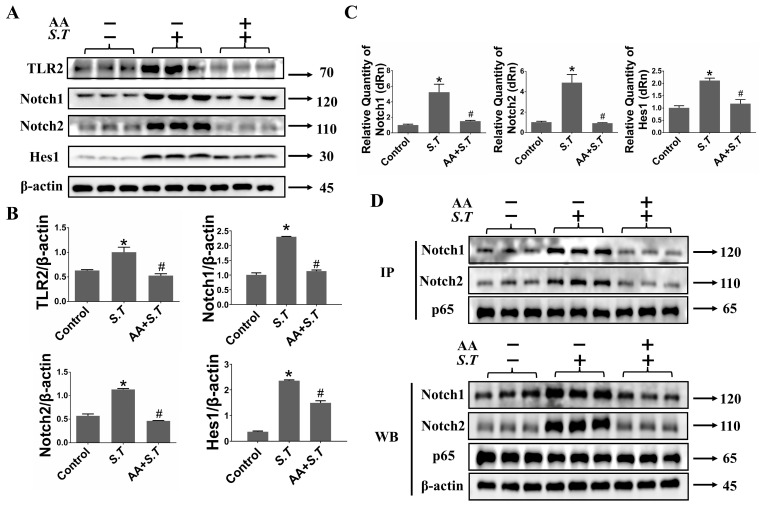
Effect of AA on *S.T*-induced TLR2/Notch pathway proteins in BV-2 cells. (**A**) Activation of TLR2/Notch pathway in BV-2 cells after *S.T* infection and AA pretreatment detected by Western blot. (**B**) TLR2/Notch pathway protein grayscale images in BV-2 cells (*n* = 3, * *p* < 0.05, compared with control; # *p* < 0.05, compared with *S.T*). (**C**) Determination of *Notch1*, *Notch2* and *Hes1* mRNA expression after *S.T* infection and pretreatment with AA by qPCR in microglia (*n* = 3, * *p* < 0.05, compared with control; # *p* < 0.05, compared with *S.T*). (**D**) Co-IP determination of protein interactions between Notch and p65 after *S.T* infection and AA pretreatment in microglia.

TLR2, abundantly expressed in microglia, plays a critical role in immune response initiation. TLR2 promotes neurotoxic inflammatory responses in microglia, mediating neuronal death [39]. We examined the changes in TLR2 downstream Notch pathway in BV-2 cells after *S.T* infection and AA pretreatment. The results revealed that TLR2 expression increased after *S.T* infection compared with control (Figure 5A,B). AA pretreatment significantly reduced *S.T*-induced TLR2 activation (Figure 5A,B). Inhibition of the Notch pathway in BV-2 cells reduces neural damage [40]. Western blot and qPCR assays of the Notch pathway revealed that AA interfered with *S.T*-induced activation of the Notch pathway in BV-2 cells (Figure 5A,C). These results demonstrated that the TLR2/Notch pathway is inhibited by AA upon *S.T* stimulation.

The Notch transmembrane domain NICD activates phosphorylation of p65 by binding the ANK domain to p65, thereby regulating the activation state of intradomain pathways [22]. In the present study, p65 phosphorylation was altered by *S.T* infection and AA pretreatment; however, the interaction between Notch and p65 required further clarification. Therefore, we performed co-immunoprecipitation assays using a p65 antibody. Notch1 and Notch2 were detected in the p65 immunoprecipitated complexes. *S.T* infection increased the levels of p65-associated Notch1 and Notch2 relative to controls, whereas AA pretreatment reduced the *S.T*-induced interactions of Notch1 and Notch2 with p65 (Figure 5D).

### 2.7. TLR2 Positively Regulates Notch Pathway in S.T Infection in BV-2 Cells

**Figure 6:**
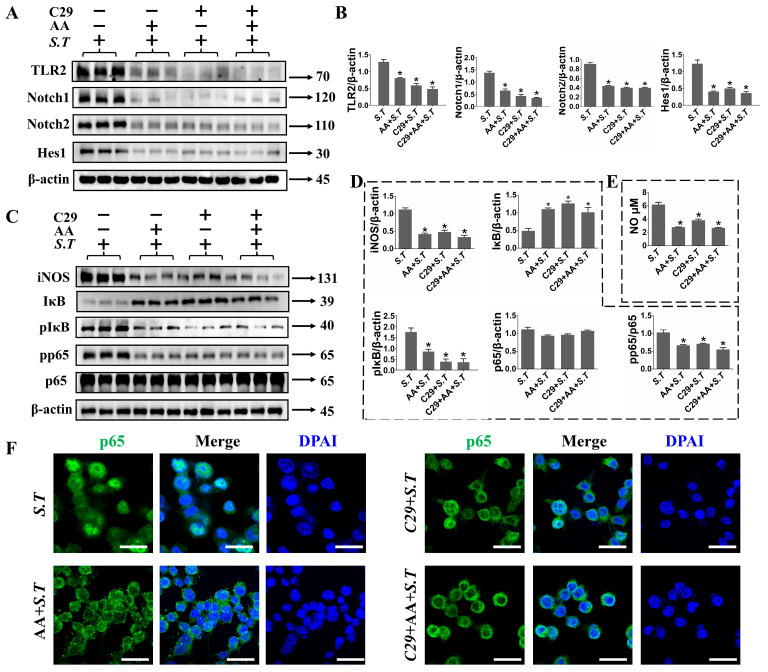
Inhibition of the TLR2 facilitates reduction in Notch, iNOS and NF-κB activity in *S.T*-infected BV-2 cells. (**A**) Western blot to detect Notch pathway protein changes in *S.T* infection BV-2 cells after TLR2 inhibition. (**B**) Grayscale values of Notch pathway protein bands in *S.T*-stimulated BV-2 cells after TLR2 inhibition (*n* = 3, * *p* < 0.05, compared with control; *p* < 0.05, compared with *S.T*). (**C**) Activation of iNOS and NF-κB pathway in *S.T*-infected microglia by Western blot after TLR2 inhibition. (**D**) Grayscale values of iNOS and NF-κB pathway protein bands in microglia after TLR2 inhibition (*n* = 3, * *p* < 0.05, compared with control; *p* < 0.05, compared with *S.T*). (**E**) NO levels in the supernatant of *S.T*-infected microglia after TLR2 inhibition (*n* = 3, * *p* < 0.05, compared with control; *p* < 0.05, compared with *S.T*). (**F**) Confocal representative images demonstrate p65 nuclear translocation in microglia after TLR2 inhibition (scale = 20 μM).

The TLR2/Notch pathway is activated to promote inflammatory factor release in *S.T*-infected microglia. To determine whether TLR2-mediated signaling enhanced Notch, a TLR2 inhibitor C29 were used in BV-2 cells to check Notch1 pathway proteins expression. As noted in Figure 6A,B, C29 reduced *S.T*-induced expression of Notch1, Notch2 and Hes1. We have previously known that p65 nuclear translocation promotes transcription by binding to NICD, so Notch expression is reduced after TLR2 blockade, NF-κB activity should be diminished accordingly. After confirming the features of TLR2-mediated activation of Notch1 signaling, we investigated its role in the TLR2 canonical pathway for activation of NF-κB in microglia. As we suspected, C29 reduced *S.T*-stimulated NF-κB activation as manifested by reduced IκB phosphorylation and reduced p65 phosphorylation and nuclear translocation (Figure 6C,D,F). The p65 in microglia binds to the nuclear DNA and promotes the transcription of iNOS, which releases large amounts of NO. As observed in Figure 6C–E, pretreatment with C29 prior to *S.T* infection resulted in reduced iNOS protein expression and NO release in microglia. All these results demonstrate that TLR2 mediated activation of the Notch pathway in *S.T* stimulation leads to increased NF-κB mediated gene function in microglia.

**Figure 7:**
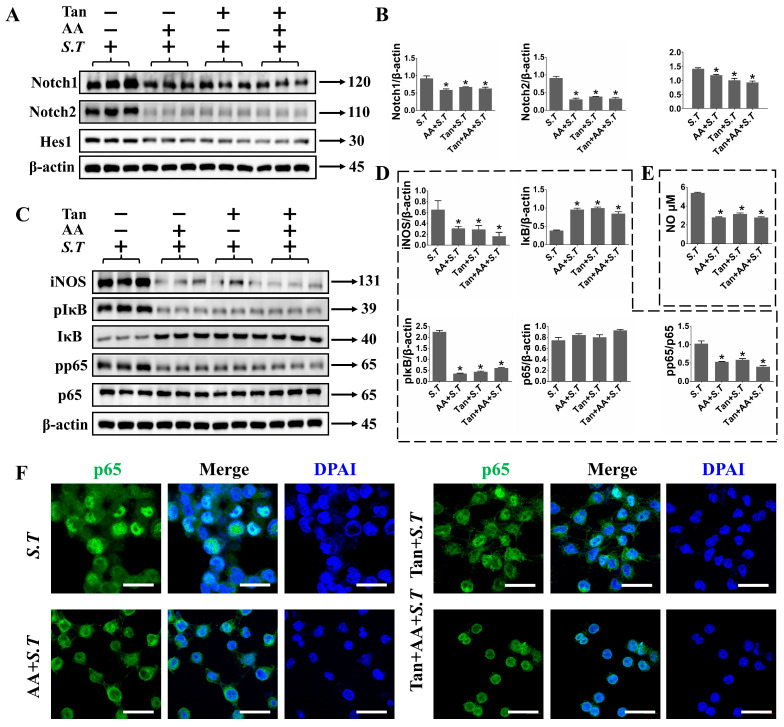
Inhibition of the Notch pathway facilitates reduction in iNOS and NF-κB activity in *S.T*-infected BV-2 cells. (**A**) Western blot to detect Notch pathway proteins changed in *S.T* infection BV-2 cells after Notch inhibition. (**B**) Grayscale values of Notch pathway protein bands in *S.T*-stimulated BV-2 cells after Notch inhibition (*n* = 3, * *p* < 0.05, compared with control; *p* < 0.05, compared with *S.T*). (**C**) Activation of iNOS and NF-κB in *S.T*-infected BV-2 cells by Western blot after Notch inhibition. (**D**) Grayscale values of iNOS and NF-κB pathway protein bands in BV-2 cells after Notch inhibition (*n* = 3, * *p* < 0.05, compared with control; *p* < 0.05, compared with *S.T*). (**E**) NO levels in the supernatant of *S.T*-infected BV-2 cells after Notch inhibition (*n* = 3, * *p* < 0.05, compared with control; *p* < 0.05, compared with *S.T*). (**F**) Confocal representative images demonstrate p65 nuclear translocation in BV-2 cells after Notch inhibition (scale = 20 μM).

The NICD seems to be a pivotal regulator of NF-κB signaling pathways, which facilitates subsequent phosphorylation and activation of IκB ubiquitinoylation through interaction with the p65 and promotes p65 nuclear translocation for NF-κB-mediated gene transcription. The results showed that Notch inhibitor (Tan) inhibited the expression of Notch1, Notch2 and Hes1 (Figure 7A,B). Phosphorylation and nuclear translocation of p65 and phosphorylation of IκB were prevented after pretreatment with Tan, indicating that activation of p65 by *S.T* is dependent on Notch activation (Figure 7C,D,F). The expression of NO and iNOS decreased following inhibition of Notch1 (Figure 7C,E). These results suggest that in *S.T*-infected microglia, the synergistic interaction between TLR2 and Notch promotes high iNOS expression through activation of downstream NF-κB.

## 3. Discussion

Growing evidence suggests that neuroinflammation and CNS disorders are closely related [41,42,43]. *S.T* penetrates the blood–brain barrier to cause meningitis, while releasing large amounts of NO and inflammatory factors [44,45]. High levels of NO lead to mitochondrial dysfunction and increased ROS secretion that promotes neuronal cell death [46]. Our results show that highly aggregated NO and mitochondrial injury in mice hippocampus after *S.T*-infected. It has been shown that of the multiple NOS isoforms that can catalyze NO synthesis, iNOS is most associated with antimicrobial activity [47]. Activation of downstream NF-κB pathway by iNOS-derived NO exacerbates inflammatory response [48]. Consistent with previous studies, we found that that excess iNOS induced high concentrations of NO in the *S.T*-infected hippocampus. Loss of iNOS reduces local inflammation; we thus inhibited iNOS expression before *S.T*-induced by selective inhibitor 1400W [49]. It was found that NO levels in the hippocampus of mice treated with 1400W were not abnormal and mitochondrial damage in the hippocampus was alleviated to some extent. Moreover, microglia regulated the secretion of NO in brain to produce neurotoxicity [50], and we also found that iNOS is highly expressed in the hippocampus near microglia. In vitro culture of BV-2 cells revealed that NO and iNOS were significantly elevated after *S.T* infection. All these results indicating that increased levels of NO derived by iNOS is the main factor in CNS infections caused by *S.T*. As a natural extract that relieves neuroinflammation, AA exhibited excellent inhibitory effects on iNOS and NO in hippocampus and BV-2 cells, and alleviated mitochondrial damage, confirming that AA inhibits *S.T*-induced damage by inhibiting the expression of iNOS. In conclusion, AA inhibited NO production through repressing of iNOS expression in BV-2 cells and hippocampus infected by *S.T*.

The expression of iNOS is dependent on TLRs activation. Activation of TLR4 promotes iNOS expression and enables cells to secrete more NO to promote bacterial infection [51]. In this study, we found elevated TLR2 and iNOS expression in the hippocampus of *S.T*-stimulated mice, so we speculate that TLR2 promotes iNOS expression as an upstream. TLR^−/−^ mice have lower iNOS expression under stress, which is accompanied by lower amounts of NO and inflammatory responses [52]. AA reduces *S.T*-induced iNOS expression in the hippocampus. However, although TLR2^−/−^ mice exhibited decreased cytokine storm and extended survival times in *S.T* infections, but induced high expression of iNOS due to higher levels of TLR4 [53]. This suggests that iNOS expression may not be entirely dependent on alterations in TLR2, and it is unclear whether iNOS in *S.T* infection is directly related to TLR2. Host-initiated defense responses generally involve TLR receptor recognition on leukocytes to proceed, and we found that BV-2 cells are activated after *S.T* infection. We analyzed the relationship between TLR2 and iNOS in BV-2 cells, to verify the iNOS expression in *S.T* infection is dependent on TLR2 activation. Results showed that iNOS expression is reduced when TLR2 is inhibited, and it is indicated that AA blocked *S.T* damage to the CNS through inhibition of TLR2-regulated iNOS expression.

Notch pathway is involved in the transmission of TLR2 to downstream pathways. In recent years, activation of the Notch pathway has been frequently reported in cancer therapy [54], but the role of inhibiting the activation of the Notch pathway in regulating nerves should not be overlooked. Notch2 has been reported to be a major metabolite in Parkinson’s disease depression [55]. Silencing Notch1 can inhibit the proliferation of glioma cells and promote the autophagy of glioma cells [56]. Targeted inhibition of the Notch1 modulates microglial inflammation and decreased the expression of pro-inflammatory factors IL-1β, and TNF-α in focal cerebral ischemia [57]. Lycopene alleviates chronic stress-induced spleen apoptosis and immunosuppression via inhibiting the Notch pathway in rats [13]. However, the Notch pathway is essential for T cell development, and deletion of Hes1 reduces the developmental efficiency of T cells [58]. In APP/PS1 transgenic mice, upregulation of Notch1 and Hes1 alleviates cognitive impairment [59]. The up-regulation and down-regulation of Notch seem to have the opposite effect on the different diseases. Our study found that the Notch pathway was activated during *S.T* infection, and the expression of its downstream protein Hes1 was increased, while the Notch pathway was inhibited and the downstream protein Hes1 expression decreased after AA pretreatment. NF-κB phosphorylation and nuclear translocation activation were attenuated after the use of Notch inhibitor Tan. Moreover, Tan also inhibited *S.T* activation of the NO and iNOS in BV-2 cells, all of which confirm the important role of Notch in *S.T* infection and AA pretreatment.

As a downstream of the Notch pathway, NF-κB is present in almost all types of animal cells, and involved in cellular responses to stimuli, including regulating the expression of various cytokines, chemokines, immune receptors, growth factors, acute phase proteins and adhesion molecules. In diabetic rats, promoting activation of NF-κB exacerbates inflammation and apoptosis in the hippocampus [60]. Reports have used natural products to inhibit the activation of NF-κB pathway to alleviate the disease process [61,62]. In this study, AA inhibited the phosphorylation and translocation of p65 caused by *S.T* infection, demonstrating that AA can reduce inflammation by inhibiting the activation of the NF-κB pathway. In addition, we found protein interactions between p65 and Notch, suggesting that NF-κB acts as and transcription factor that facilitates TLR2/Notch pathway transduction. In conclusion, AA inhibited the NF-κB pathway and attenuated the damage caused by *S.T*.

In this study, we simulated a mouse model of natural *S.T* infection with oral administration of and identified iNOS-induced elevated NO expression as an important pathway for *S.T* damage to mouse hippocampal mitochondria. At the same time, the BV-2 cells were activated, and TLR2 and its downstream pathway parade including Notch, NF-κB were elevated after *S.T* infection. Mitochondrial damage and BV-2 cells activation are intervened by AA pretreatment. Meanwhile, AA pretreatment inhibited *S.T*-induced elevated iNOS and NO expression, TLR2 and its downstream signals Notch and NF-κB, suggesting that AA may serve as a potential inhibitor for the treatment of neurological injury.

## 4. Materials and Methods

### 4.1. Antibodies

The following antibodies were used: anti-p65 (#8242, Cell Signaling Technology, Danvers, MA, USA), anti-phospho-p65 (#3033, Cell Signaling Technology), anti-IκB (#76041, Cell Signaling Technology), anti-phospho-IκB (#2859, Cell Signaling Technology), anti-Notch1 NICD (#3608, Cell Signaling Technology), anti-Notch2 NICD (#5732, Cell Signaling Technology), anti-Iba1/AIF-1 (#17198, Cell Signaling Technology), Hes1 (#11988, Cell Signaling Technology), iNOS (18985, Proteintech, Wuhan, China) anti-β-actin (66009, Proteintech), Goat anti-Mouse IgG (H+L)-HRP (PR30012, Proteintech), Goat anti-Rabbli IgG (H+L)-HRP(PR30011, Proteintech), Goat anti-Rabbit lgG AF 488 (M21012, Abmart, Shanghai, China), Goat anti-Mouse lgG AF 594 (M21013, Abmart).

### 4.2. Animals

All animal experiments were approved by the Experimental Animal Ethics Committee of Lanzhou Institute of Husbandry & Pharmaceutical Sciences of CAAS [permission number: SYXK(Gan) 2019-0002] and were performed under strict supervision. It has been reported that female mice are more sensitive to terpenoids, so female mice were selected for this experiment [63]. BALB/c mice female aged 6–8 weeks were purchased from the experimental animal center of Lanzhou Institute of Veterinary Medicine of CAAS and housed in a temperature (23 ± 2 °C) and light (12 h light/dark cycle) controlled room with ad libitum access to food and water. The mice in the control group (*n* = 6) ingested 0.9% saline by intragastric administration (i.g) on 1–14 days. Mice in *S.T* group (*n* = 6) were given with 0.9% saline on 1–7 days, then 1 × 10^9^ CFU *S.T* (i.g) on 8–14 days. Mice in AA+ *S.T* group (*n* = 6) ingested 10 mg/kg AA on 1–7 days, then 1 × 10^9^ CFU *S.T* (i.g) on 8–14 days. Mice in the 1400W + *S.T* group (*n* = 6) were injected intraperitoneally with iNOS inhibitor 1400W (20 mg/kg, Code No. M10446, AbMole, Houston, TX, USA) for three days before infection, followed by *S.T* infection for 7 days. Mice were sacrificed on day 15 and brains were removed for subsequent experiments.

### 4.3. S.T and AA Act on BV-2 Cells

The immortalized BV-2 cell line (Procell life Science & Technology, Wuhan, China) was used in vitro assays, as it possessed the morphological, phenotypic and functional characteristics of primary cultured BV-2 cells. Cells were cultured at 37 °C in a 5% (*v*/*v*) CO_2_ atmosphere. Cells were treated with or without 12.5 μM AA for 1 h, then infected with *Salmonella typhimurium* (*S.T*) SL1344 (ATCC14028, National Center for Medical Culture Collections, Beijing, China) multiplicity of infection = 100 for 24 h. Notch1 inhibitor Tangeretin (Tan) (Abmole) and TLR2 inhibitor TRL2-IN-C29 (Abmole) were added to cells for 4 h before AA or *S.T* treatments.

### 4.4. TEM

Hippocampi fixed in 2.5% glutaraldehyde. (1) Dehydration: The hippocampi after pruning is carefully cleaned with tap water first. Then, 1% osmic acid was fixed at room temperature for 2 h, ethanol gradient dehydration at room temperature, 30% ethanol, 50% ethanol, 70% ethanol, 80% ethanol, 95% ethanol, 100% ethanol I and 100% ethanol II for 20 min, 100% acetone I and 100% acetone II for 15 min. (2) Embedding: pure acetone + embedding solution (2:1) was incubated for 3 h at room temperature, pure acetone + embedding solution (1:2) was incubated overnight at room temperature, and the pure embedding solution was embedded at 37 °C for 2 h. (3) Curing: overnight in an oven at 37 °C, standing in an oven at 45 °C for 12 h, and standing in an oven at 60 °C for 24 h. (4) Ultrathin microtome (UC7, Leica, Wetzlar, Germany) sliced 50 nm, 3% uranyl acetate-lead citrate double staining, observed by transmission electron microscope. Simples were observed with a TEM (HT7800, HITACHI, Tokyo, Japan).

### 4.5. IHC

The mouse brain was fixed with 4% paraformaldehyde, dehydrated with ethanol gradient and embedded with wax. Each embedded wax block was sliced with a thickness of 6 μm, dewaxed, EDTA antigen repaired 100, boiled for 20 min, then treated with 2% hydrogen peroxide, and closed with bovine serum albumin for 1 h. Sections were incubated with primary and secondary antibodies, respectively, followed by staining with DPAI (Beyotime, Shanghai, China). Images were observed with a TEM (HT7800).

### 4.6. NO Assay

The Griess reaction quantifies nitrite accumulation in the hippocampus and culture medium as an indicator of NO production. In vivo experiments, hippocampal samples from each group of mice underwent homogenization with PBS, followed by centrifugation at 3500 rpm for 10 min. The resulting supernatant was utilized for NO detection. In vitro studies involved direct NO detection in BV-2 cells culture medium following the manufacturer’s instructions (Beyotime). Briefly, 50 μL of Griess Reagent I and 50 μL of Griess Reagent II were added to each sample, then incubated at room temperature for 20 min. Subsequently, absorbance was measured at 450 nm using the Micro-Plate spectrometer SYNERGY LX (Gene Company Limited, Hong Kong).

### 4.7. RNA Extraction and Quantitative Reverse Transcription PCR (RT-qPCR)

Total microglial and hippocampus RNA was isolated using TRIzol (Invitrogen Life Technologies, Waltham, MA, USA). mRNA was synthesized using a reverse transcription (RT) reagent kit, with gDNA Eraser (Takara, Dalian, China). Quantitative (q)-PCR analysis was performed using TB Green^®^ Premix EX Taq™ II (Takara). The cycling conditions were as follows: 95 °C for 30 s, followed by 40 cycles of 95 °C for 5 s, 60 °C for 30 s in QuantStudio 5 (Thermo Fisher Scientific, Waltham, MA, USA). Relative mRNA abundance was calculated using the 2^−ΔΔCt^ method. The gene-specific oligonucleotide primers used for qPCR: *β-actin* F-CCACCATGTACCCAGGCATT, *β-actin* R-AGGGTGTAAAACGCAGCTCA; *Notch1* F-GTGCTGGAAGTATTTTAGCGAC, *Notch1* R-GTCCTTGCAGTACTGGTCATAC; *Notch2* F-CATCAACAACCAGTGTGATGAG; *Notch2* R-TTTGTCATACTTGCACGTCTTG; *Hes1* F-AATTTGCCTTTCTCATCCCCAA; *Hes1* R-GAAGGTGACACTGCGTTAGG.

### 4.8. Immunoprecipitation (IP)

Classic Magnetic Protein A/G IP/Co-IP Kit YJ201 (Epizyme Biomedical Technology, Shanghai, China) used for IP detection. All operations are carried out according to the guide. Briefly, 25 µL IP A/G beads from each sample were rinsed three times with lysis buffer. Anti-p65 was incubated with IP protein A/G beads at 4 °C overnight. A total of 25 µL IP A/G beads per sample were rinsed three times with lysis buffer. Lysate of BV-2 cells were incubated with IP beads at 4 °C overnight. IP beads were rinsed three times with lysis buffer. Five-fold loading buffer was added to the sample. The samples were boiled at 100 °C for 10 min, and collected the supernatant and removed tube on the magnetic for 2 min. The supernatant was stored −20 °C for Western blots. The antibodies for Western blot were anti-p65, anti-Notch1 and anti-Notch2.

### 4.9. Western Blot

The whole protein of BV-2 cells and hippocampus was extracted by whole cell lysis assay (Thermo Fisher Scientific), including protease inhibitor, PMSF and phosphatase inhibitor (KeyGene Biotech, Nanjing, China). BV-2 cells and brain were lysed and waited on ice for 5 min, then vigorously shaken for 30 s, repeated 5 times, centrifuged at 12,000× *g* for 15 min, and the supernatant was taken. The protein concentration was detected by BCA protein assay kit (Beyotime), and 5× loading buffer was added, then boiled at 100 °C for 5 min for subsequent experiments. The sodium dodecyl sulfate polyacrylamide gel electrophoresis (SDS-PAGE) was electrophoresed at 150 V for 60 min, and then transferred to the membrane at 200–300 mA for 70 min. Mouse antibody (4 °C overnight) and horseradish peroxidase-labeled (HRP) goat anti-rabbit or mouse IgG (37 °C for 1 h) were incubated, respectively. ImageJ 1.53t (Wayne Rasband, NIH, Bethesda, MD, USA) softwarewas used to determine densitometric values of immunoblot signals from three separate experiments.

### 4.10. IF

Cells were fixed with 4% paraformaldehyde for 20 min, blocked with 5% BSA (Beyotime) for 1 h, then hybridized with specific antibodies against p65 (Cell Signaling Technology) at 4 °C. They were then incubated in the dark for 1 h with anti-mouse IgG secondary antibody AF 488 Conjugate, then stained with DAPI (Beyotime) in the dark for 5 min. Cells were finally observed with a laser confocal microscope.

### 4.11. CCK8

BV-2 cells were cultured in 96-well plates. After stimulating the cells with specified doses of AA and *S.T*, respectively, 10 µL of CCK-8 (Beyotime) reagent was added to each well. Thirty minutes later, the optical density (OD) of the cells was measured at 450 nm.

### 4.12. Mitochondrial Function Assay

Freshly isolated mouse hippocampal tissue underwent mitochondrial extraction following the manufacturer’s protocol (KeyGEN BioTECH, Nanjing, China). Briefly, the process involved grinding the tissue on ice in pre-cooled Mito Lysis Buffer, adding Medium Buffer, and isolating mitochondria through multiple centrifugation steps. The mitochondrial precipitate was then washed with PBS, resuspended in HBSS, and plated onto 96-well plates. Subsequently, each well received 10 μL of BBoxiProbe^®^ R02 oxygen fluorescent probe (BestBio, Beijing, China) and 100 μL of oxygen blocking solution (BestBio). Relative Fluorescence Units (RFU) were recorded every 3 min for 5 measurements (totaling 15 min) using an excitation wavelength of 468 nm and an emission wavelength of 603 nm. The OCR is calculated as the change in RFU divided by the change in time, specifically, the difference between the RFU at the end point and the start point over the corresponding time interval.

The mitochondrial membrane potential of BV-2 cells was measured using a specific assay kit (BestBio). Briefly, mitochondria were incubated with the Rhodamine 123 probe for 15 min. Following the manufacturer’s instructions, the RFU was then measured with a microplate reader at an excitation wavelength of 490 nm and an emission wavelength of 530 nm.

### 4.13. Statistical Analysis

Statistical analysis was performed using SPSS software (version 20.0). All images and data were analyzed in the double-blind manner and random sampling. Data are expressed as the mean ± standard error of mean (SEM), and multi-group comparisons of the means were performed using one-way ANOVA analysis. A difference with *p* < 0.05 was considered statistically significant.

## Data Availability

The raw data supporting the conclusions of this article will be made available by the authors on request.
